# Coinfection of COVID-19 and Tuberculosis in Uganda

**DOI:** 10.4269/ajtmh.22-0738

**Published:** 2023-04-24

**Authors:** Edwin Nuwagira, Stellah G. Mpagama, Asumpta Katusiime, Bobson Natamba, Joseph Baruch Baluku, Peggy S. Lai

**Affiliations:** ^1^ Department of Internal Medicine, Mbarara University of Science and Technology, Mbarara, Uganda;; ^2^ Tuberculosis Treatment Unit, Mbarara Regional Referral Hospital, Mbarara, Uganda;; ^3^ Infectious Diseases Unit, Kibong’oto Infectious Disease Hospital, Kilimanjaro, Tanzania;; ^4^ Division of Pulmonology, Kiruddu National Referral Hospital, Kampala, Uganda;; ^5^ Division of Pulmonology, Massachusetts General Hospital, Boston, Massachusetts

## Abstract

The clinical features and outcomes of tuberculosis (TB) and COVID-19 coinfection are not well established. This short report describes 11 people with TB/COVID-19 coinfection in Uganda. The mean age was 46.9 ± 14.5 years; eight (72.7%) were male and two (18.2%) were coinfected with HIV. All patients presented with cough whose median duration was 71.1 (interquartile range, 33.1, 109) days. Eight (72.7%) had mild COVID-19 whereas two (18.2%) died, including one with advanced HIV disease. All patients were treated with first-line anti-TB drugs and adjunct therapy for COVID-19 using national treatment guidelines. This report presents the possibility of the coexistence of the two diseases and calls for more vigilance, screening, and collective prevention measures for both COVID-19 and TB.

Before the COVID-19 pandemic, tuberculosis (TB) was the leading cause of death globally from a single infectious agent.^1^ However, for the last two decades, the fight against TB based on case surveillance, testing, and treatment has saved up to 66 million people worldwide,^1^ and to date, TB remains a preventable and curable disease. The two diseases, TB and COVID-19, are both transmitted through aerosols,^2^ and a dysregulated immune response to infection is what leads to host tissue damage. This suggests that people with TB/COVID-19 coinfection could potentially experience more severe disease and mortality resulting from a synergistic worsening of either disease.^3^ Moreover, traditional factors associated with TB such as low socioeconomic status, diabetes, and HIV infection, especially in immigrant populations, are also associated with severe forms of COVID-19.^4^

COVID-19 can occur before, simultaneously with, or after TB.^5^ The risk posed by latent, active, and previous TB in developing COVID-19 is yet to be clearly described, but a coinfection has been associated with fatal outcomes. The risk of TB death in COVID-19 patients is estimated to be 1.4 times higher than in people with only TB and may be higher in some populations.^5^ A study on 69 patients from eight countries found a case fatality of 11.6% (8/69) with mortality more likely to occur in elderly patients.^6^ Another study from the Philippines showed that coinfection of COVID-19 and TB raised the odds of mortality by 2.17 times, and the same was reflected in a South African study.^7^

Uganda’s high TB burden is ranked among the world’s top-30 high-TB-burden countries.^1^ Patients with COVID-19 in Uganda, like elsewhere in the world, present with respiratory symptoms such as fever, cough, and shortness of breath and the mortality is higher for those admitted with severe disease.^8^

Although COVID-19 symptoms develop over a shorter period than TB, these symptoms are generally similar, making the two diseases a “cruel duet” that cannot be easily distinguished clinically. Therefore, patients who present after several days or months of respiratory symptoms and in clinical deterioration should be tested for both COVID-19 and TB.^9^ Generally, there is scant information from sub-Saharan Africa focusing on the clinical characteristics, treatment options, and treatment outcomes of patients coinfected with COVID-19 and TB. In this short report, we describe the clinical presentation, laboratory findings, and treatment outcomes of people with TB/COVID-19 coinfection in a high-TB/HIV-burden setting.

In an ongoing cohort study enrolling adults with TB at Mbarara Regional Referral Hospital in Uganda, all patients with confirmed TB were screened for COVID-19 before admission to the TB treatment unit or enrollment in our study during the SARS-CoV-2 Omicron subvariant surge (October 2021 to May 2022). The COVID-19 diagnosis in all patients presented in this report was made using both an antigen-based rapid diagnostic kit (Abbott Panbio^®^ COVID-19 antigen rapid test device, Lake Forest, IL) and reverse-transcriptase polymerase chain reaction assay done on a respiratory specimen at the Central Public Health Laboratory in Kampala, Uganda’s capital.

In addition to the COVID-19 test, all patients in our cohort had a chest X-ray and a complete blood count completed, liver enzymes (alanine transaminase and aspartate transaminase), kidney function (serum urea and creatinine), HIV serology, and glycated hemoglobin. We also recorded baseline anthropometric measurements, physical examination findings, and vital signs in an electronic data capture tool (REDCap software; Harvard University, Boston, MA). We compared baseline clinical characteristics, laboratory values, and 2-week outcomes of participants who had only TB and those coinfected with both TB and COVID-19. Ethical approvals for the study were provided by the Mbarara University of Science and Technology (MUST) Research and Ethics Committee (MUST-2021-61) and the Uganda National Council of Science and Technology (HS1579ES).

As of June 2022, 37 patients with pulmonary TB confirmed by Xpert MTB/RIF Ultra (Xpert Ultra; Cepheid, Sunnyvale, CA) had been enrolled, and out of these 11 (29.7%) had a positive test for COVID-19. Two of the COVID-19-positive individuals were also HIV infected. Only three patients of the 11 were hospitalized whereas the remaining eight were managed with home-based care. The main symptoms and laboratory values are shown in Table 1. Those admitted had unstable vital signs, and their chest X-rays are shown in Figure 1. One of the admitted patients, an 18-year-old male, was transferred from the COVID-19 treatment center to the TB unit. Having spent 10 days in the COVID-19 treatment center, the diagnosis of TB was suspected based on persistent fevers, cough, and worsening difficulty in breathing on a background of advanced HIV disease. Mycobacterium tuberculosis (MTB) was identified in his sputum using Xpert Ultra. This patient died after 2 days of admission to the TB unit. Due to the nature and clinical presentation of the disease, COVID-19 was diagnosed first in this patient and TB was diagnosed later through radiography and Xpert Ultra due to persistent symptoms. The second admitted patient, a 40-year-old male who was HIV negative, developed difficulty in breathing but died shortly after being initiated with a high flow of oxygen. The circumstances surrounding his death were not clear and no autopsy studies were done. The third admitted patient, a 28-year-old male of Asian descent, presented with fevers, weight loss, and difficulty in breathing. He improved on antibiotics and oxygen and was still in the hospital after 14 days of admission. None of the three critically ill patients had any other chronic medical conditions apart from the patient who had advanced HIV disease. Overall, four patients had a history of smoking and only two of the 11 had received at least one dose of the COVID-19 vaccine.

**Table 1:** Baseline characteristics

Characteristic	Overall	TB only	TB/COVID-19 coinfection	*P* value
No. of people, *n* (%)	37	26 (70.3)	11 (29.7)	
Age, mean ± SD, years	43.6 ± 14.8	42.3 ± 15	46.9 ± 14.5	0.37
Male sex, *n* (%)	26 (70.3)	18 (69.2)	8 (72.7)	0.83
BMI, median (IQR), kg/m^2^	18.9 (17.4, 20.3)	18.8 (16.9, 20.6)	19.15 (16.8, 21.5)	0.6
MUAC, median (IQR), cm	23.4 (22.6, 24.2)	23.2 (22.3, 24.2)	24.1 (22.3, 25.7)	0.82
HIV infected, *n* (%)*	9 (24.3)	7 (26.9)	2 (18.2)	0.32
Systolic blood pressure, mm Hg (IQR)	113.9 (108.5, 119.3)	114.5 (108.6, 120.3)	112.6 (99.1, 126.1)	0.37
History of TB, *n* (%)	3 (8.1)	3 (11.5)	0	0.24
COVID-19 vaccination, *n* (%)	9 (24.3)	7 (26.9)	2 (18.1)	0.57
Smoking history, *n* (%)	13 (35.1)	8 (61.5)	5 (38.5)	0.39
Alcohol intake, *n* (%)	26 (70.3)	19 (73.1)	7 (26.9)	0.56
Symptoms, *n* (%)				
Fever	33 (89.2)	23 (88.5)	10 (90.9)	0.82
Cough	37 (100)	26 (100)	11 (100)	
Hemoptysis	3 (8.11)	3 (11.5)	0	0.24
Chest pain	28 (75.7)	19 (73.1)	9 (81.8)	0.57
Duration of symptom, median (IQR), days				
Fever	48.5 (25.5, 71.6)	44.5 (13, 75.8)	57.8 (25.3, 90.72)	0.59
Cough	62.7 (41.1, 84.4)	66.5 (37.3, 95.7)	53.8 (23.3, 84)	0.59
Quantitative Xpert Ultra, *n* (%)				0.72
High	14 (37.8)	12 (46.1)	2 (18.2)	
Moderate	7 (18.9)	2 (7.7)	5 (45.5)	
Low	16 (43.2)	12 (46.2)	4 (36.7)	
Laboratory values, median (IQR)				
White cell count, cells/µL	6.78 (5.7, 7.8)	6.9 (5.7, 8.1)	8.2 (6.3, 10.1)	0.31
Lymphocytes, cells/µL	1.5 (1.2, 1.7)	1.6 (1.2, 1.9)	1.2 (0.7, 1.6)	0.12
Neutrophils, cells/µL	4.4 (2.3, 6.4)	4.4 (3.2, 5.6)	4.4 (2.3, 6.4)	0.96
Platelets, cells/µL	320 (268, 371)	346 (292, 400)	262 (140, 384)	0.12
ALT, U/L	34.4 (20.1, 48.5)	32.1 (17.7, 46.6)	39 (2, 76.3)	0.64
Hb A1C, %	6.4 (5.6, 7.14)	6.4 (5.6, 7.3)	6.3 (4.5, 8.2)	0.89

*P* values are Kruskal–Wallis or χ^2^. ALT = alanine transferase; AST = aspartate transaminase; BMI = body mass index; Hb A1C = hemoglobin A1C; IQR = interquartile range; MUAC = mid-upper-arm circumference; TB = tuberculosis.

* Median CD4 in patients who were HIV infected with TB only was 146 cells per cubic millimeter, IQR (14.8, 277.1), whereas that for patients with TB and COVID-19 was 521 cells per cubic millimeter; the third patient had no CD4 count detected.

**Figure 1.**
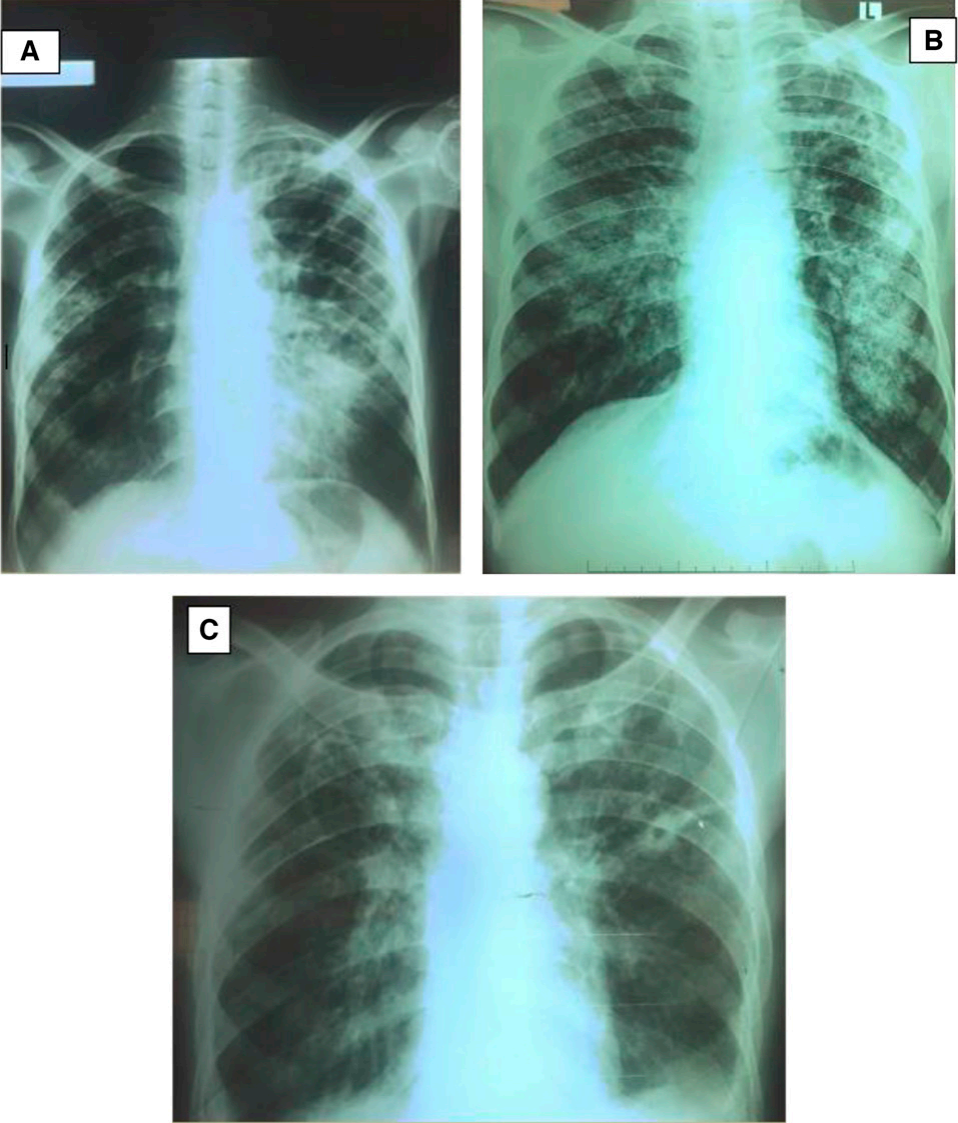
Chest radiographs of the three patients who had severe COVID-19 and tuberculosis. (**A**) Left lung consolidation and fibrosis in a 18 years old male. (**B**) Bilateral lung infiltrates in a 40-year-old male. (**C**) Hilar lymphadenopathy, right apical infiltrates and left lung cavitation in a 28-year-old male.

During admission, patient management was guided by Uganda national treatment guidelines for both diseases. Patients with TB received weight-based fixed-dose combination drugs: oral rifampicin, ethambutol, pyrazinamide, and isoniazid for 2 months, to be followed by 4 months of rifampicin and isoniazid.^10^ Patients with COVID-19 received supportive treatment, such as paracetamol for fever control as required, vitamin D, zinc, intravenous ceftriaxone 2 g daily for 1 week, and azithromycin 500 mg daily for 5 days, depending on the disease severity, as per Uganda COVID-19 treatment guidelines.^11^

At the peak of the COVID-19 pandemic in Uganda, TB remained a neglected disease with minimal efforts from the government to invest in research and discover new drugs and novel diagnostic methods. The decline in the TB case surveillance that equally affected low- and middle-income countries that have a high burden of TB has led to a rise in TB cases globally despite the previously achieved downward trend over the past 5 years. The first reported case of TB/COVID-19 coinfection was of a 26-year-old Haitian male who had multidrug-resistant TB, and since then many more cases have been reported. Although concurrent infection of MTB and SARS-CoV-2 is not unique to our setting, a recent cohort of a TB/COVID-19 coinfection report did not include data from sub-Saharan Africa, which has a high burden of TB and HIV.^12^

Tuberculosis seems predisposed to severe forms of COVID-19,^13^ but whether it is the cause of the severity in this group remains unknown. Despite the documented short duration of illness in several COVID-19 cohorts, our patients had a median duration of respiratory symptoms of 71.1 days (interquartile range, 33.1, 109 days). Our patients probably had TB and were superinfected with COVID-19, which worsened their respiratory symptoms and prompted them to seek health care. This further explains the unmet need for the timely diagnosis and treatment of TB in high-TB-burden countries. Currently, there no guidelines for the treatment of TB/COVID-19 coinfection. Although corticosteroids were approved for the management of severe forms of COVID-19^14^ and some forms of TB,^15^ there is no evidence of their benefit in cases with sputum positive for TB coinfected with COVID-19. Additionally, given the multiple drugs given to patients with both diseases, there is a need to monitor for drug interactions that could potentially lead to fatal outcomes.

This information gives a snapshot of what may be happening in other low-income, high-TB-burden settings where screening for COVID-19 is not routinely done for patients diagnosed with TB. We also recommend that testing and treatment guidelines be tailored to the management of both pulmonary diseases to improve patient outcomes. Despite the low numbers in our study, to our knowledge this is the first study from Uganda to report TB/COVID-19 coinfection and will be a basis for dual screening at both out- and inpatient TB and COVID-19/special pathogens clinics. We were unable to do full genome sequencing, which limited our understanding of the COVID-19 variants of our patients. However, using the country’s epidemiological data and a few sampled specimens, the majority of the infections at the time of enrollment of our participants were from the Omicron subvariant.^16^ In conclusion, there is a need for a data-sharing platform for resource-limited settings that provides country-level information to be used to describe the epidemiology of TB and COVID-19 coinfection, optimize diagnostic and treatment guidelines, and understand how TB/COVID-19 coinfection subsequently affects lung function.

## Financial Disclosure

This work is funded by a Multimorbidity Research Capacity Initiative Grant at the Mbarara University of Science and Technology sponsored by the NIH (D43TWO11632) and an American Thoracic Society Diversity Grant.
